# Retraction Note: Annexin A1-suppressed autophagy promotes nasopharyngeal carcinoma cell invasion and metastasis by PI3K/AKT signaling activation

**DOI:** 10.1038/s41419-025-07540-0

**Published:** 2025-03-19

**Authors:** Jin-Feng Zhu, Wei Huang, Hong-Mei Yi, Ta Xiao, Jiao-Yang Li, Juan Feng, Hong Yi, Shan-Shan Lu, Xin-Hui Li, Rou-Huang Lu, Qiu-Yan He, Zhi-Qiang Xiao

**Affiliations:** 1 Research Center of Carcinogenesis and Targeted Therapy, Xiangya Hospital, Central South University, 410008 Changsha, Hunan China; 2 Department of Gastrointestinal Surgery, The Affiliated Cancer Hospital of Xinjiang Medical University, 830011 Urumqi, Xinjiang China; 3 Institute of Dermatology, Chinese Academy of Medical Sciences and Peking Union Medical College, 210042 Nanjing, Jiangsu China; 4 Department of Pathology, Xiangya Hospital, Central South University, 410078 Changsha, Hunan China

Retraction Note to: *Cell Death & Disease* 10.1038/s41419-018-1204-7, published online 20 November 2018

The Editors-in-Chief have retracted this article. Concerns have been raised regarding Figures 4a and Figure 5a, b and c, specifically:

Figure 4a: the panel for 0h 5-8F Blank appears to partially overlap with the panel for 0h 5-8F Scr shRNA.

Figure 5a: the panel for 0h 5-8F-ANXA1 KD siBECN1(50 nM) appears to be identical to the panel for 0h 5-8F-Scr shRNA 3-MA(7.5mM) of Figure 5c, the panel for 0h 5-8F-Scr shRNA siBECN1(50 nM) of Figure 5a and the panel of 0h 5-8F-Scr shRNA siNC(50 nM) of Figure 5b.

Figure 5a: the invasive cells panel for 5-8F-Scr shRNA siNC(50 nM) appears to partically overlap with the the invasive cells panel of 5-8F Scr shRNA Vehicle of Figure 5c.

Figure 5a: the invasive cells panel for 5-8F-Scr shRNA siBECN1(50 nM) appears to be identical to the invasive cells panel for 5-8F-Scr shRNA siATG5(50 nM) of Figure 5b.

The Editors-in-Chief therefore no longer have confidence in the results and conclusions reported in this article.

Authors Jin-Feng Zhu, Wei Huang, Hong-Mei Yi, Ta Xiao, Jiao-Yang Li, Juan Feng, Hong Yi, Shan-Shan Lu, Lu, Qiu-Yan He & Zhi-Qiang Xiao agree with this retraction. The publisher has been unable to obtain a current email address for authors Rou-Huang Lu and Xin-Hui Li.

